# Hand-Book of Skin Diseases

**Published:** 1865-05

**Authors:** 


### Book-Notices.

HAND-BOOK OF SKIN DISEASES, FOR STUDENTS AND PRACTITIONERS. By THOMAS HILLIER, M.D., London, Member of the Royal College of Physicians; Physician to the Skin Department of University College Hospital; Physician to the Hospital for Sick Children. With Illustrations. Philadelphia: BLANCHARD & LEA. 1865.

This is a moderate sized octavo volume, of 353 pages; neatly printed, and bound in cloth. It is a concise, plain, practical treatise on the various diseases of the skin; just such a work, indeed, as was much needed, both by medical students and practitioners. After the introductory chapter, the author briefly reviews the classifications of Galen, Mercurialis, Frak, Wilson, Hebra, and others, gives a chapter to the anatomy and physiology of the skin; another to the definition of terms; and one each to etiology and diagnosis. He then treats the various diseases in detail, under the following heads:---Exanthemata; papular diseases; squamous diseases; vesicular diseases; pustular diseases; hemorrhagic diseases; maculæ and pigmentary diseases; diseases due to changes in the sebaceous follicles; hypertrophies and degenerations; gangrenous inflammations of the skin; parasitic diseases; diseases of the nails and hairs; acute eruptive diseases; syphilides, and appendix.

For sale by W. B. Keen & Co., 148 Lake St., Chicago.

